# Prevalence of Failure to Thrive in a Tertiary Care Center in Saudi Arabia

**DOI:** 10.7759/cureus.93145

**Published:** 2025-09-24

**Authors:** Khalid Alghamdi, Faris Althubaiti, Saud Bamousa, Muath Bashanfar, Mohammed Alqarni, Hassan Alariany, Muhannad Alqarni, Ali Alnakhli, Raed Alshehri, Abdulaziz K Alsulaiman

**Affiliations:** 1 Pediatrics, King Abdulaziz University, Jeddah, SAU; 2 Pediatric Neurology, King Abdulaziz University, Jeddah, SAU

**Keywords:** developmental delay disorders, failure-to-thrive, ftt, malnutrition, newborn and child health, pediatric growth disorders, saudi arabia, underweight children

## Abstract

Background

Failure to thrive (FTT) is referred to as inadequate growth focusing on anthropometric criteria that indicate poor nutritional status and growth. Despite the importance of FTT among pediatric age groups, there are a limited number of studies in Saudi Arabia. Therefore, this study aims to determine the incidence of FTT in a tertiary care center in Jeddah, Saudi Arabia.

Methodology

This is a prospective cross-sectional study conducted at King Abdulaziz University Hospital (KAUH) in Jeddah, Saudi Arabia, between November 2023 and October 2024. The study included pediatric inpatients up to two years of age, and excluded those who did not meet the inclusion criteria. Data was collected from 142 individuals through an anonymous questionnaire.

Results

Our study included 142 pediatric inpatients up to the age of two years, with a demographic breakdown of 56.3% male and 43.7% female. Among the participants, 70.4% were Saudi nationals, while 29.6% were non-Saudi. The majority of our population was in the age group of 0 to six months (43.7%). The mean weight of the patients was 6.61 ± 2.87 kg, and the mean height was 64.49 ± 13.06 cm. FTT was identified in 44.4% of the patients. Preterm births were identified as a significant independent risk factor for FTT, and 56.1% of patients with a history of previous neonatal or pediatric Intensive Care Unit (ICU) admissions developed FTT.

Conclusion

Our study concluded a significant incidence of FTT among pediatric inpatients up to two years of age. Nearly half of our population had FTT. Notable risk factors included preterm birth and ICU admissions. These findings highlight the burden of FTT in KAUH, as well as the need for targeted interventions to address and reduce the incidence of FTT.

## Introduction

Failure to thrive (FTT) is a term that refers to inadequate growth, which is typically diagnosed in infancy. FTT definitions typically include both the distinct anthropometric criteria that define it and the clinical features of inadequate growth. The literature on defining FTT can be broadly categorized in terms of anthropometric indicators, etiological causes, and a variety of interchangeable descriptors used to define FTT. Furthermore, relevant studies agreed that all current methods of diagnosing FTT either overdiagnose or underdiagnose the condition, and a more precise diagnostic tool could be helpful clinically [1]. FTT is often characterized in ordinary clinical practice as either a weight for age that consistently falls below the 5th percentile or weight deceleration that exceeds two major percentiles on a growth chart [2]. Essential anthropometric criteria include nutritional status, growth assessment, growth chart monitoring, and child growth.

Based on the underlying causes, FTT is divided into two main categories: organic and non-organic. Organic FTT is caused by certain medical illnesses, such as gastrointestinal issues, chronic infections, metabolic or genetic diseases, endocrine disorders, and neurological conditions, that impair a child's capacity to grow [3]. These circumstances impair the body's ability to absorb nutrients, raise metabolic demands, or directly impede the growth processes. Conversely, non-organic FTT is more frequently brought on by environmental or psychosocial causes, and is the most common cause of FTT [3,4]. These causes can include poor nutrition brought on by inappropriate feeding techniques, poverty, parental neglect, or carers' mental health problems. Non-organic FTT may also result from feeding issues like as behavioural issues or sensory aversion. Both have the potential to have a major impact on a child's growth and development, and frequently a number of factors may be at play, necessitating thorough assessments and specific intervention in order to classify it.

Studies have shown that in the United States, the percentage of children presenting to a primary care center with failure to thrive could reach 10% and approximately 5% in patients who were admitted [3,5]. A study in Denmark concluded that 27% of infants met at least one of their criteria for failure to thrive [6]. A study conducted in a tertiary care hospital in Riyadh revealed that the prevalence of FTT was 26.75% [7]. Other studies, however, usually take a more sample-based approach, aiming to associate failure to thrive to certain etiologies, such as celiac disease, which was noted to be present in patients with unexpected failure to thrive [8]. Meanwhile, some studies focused on clinics that participated in studies relating to failure to thrive, usually consisting of either gastroenterology or endocrinology clinics [9].

The rate of growth is usually measured through universally accepted growth charts, such as the ones adopted by the Centers for Disease Control and Prevention (CDC) or the World Health Organization (WHO). While slight variation exists between them, the CDC recommends that patients less than two years old should follow through with the WHO growth charts, which use data from six countries [10].

The aim of the present study is to determine the incidence of failure to thrive in a tertiary care center in Saudi Arabia. Also, we tried to find the etiologies of FTT by gathering the chronic disease status of each patient 

## Materials and methods

Ethical consideration

Ethical approval has been obtained by the Research Ethics Committee in King Abdulaziz University, with the identity of the research participants remaining confidential.

Study design

The prospective cross-sectional study was carried out at King Abdulaziz University Hospital (KAUH), Jeddah, Saudi Arabia, between November 2023 and October 2024 to establish an understanding of the local incidence of failure to thrive in a tertiary care center.

Study population

The study was conducted at King Abdulaziz University Hospital in Jeddah, Saudi Arabia. This study included all inpatient children under two years. The characteristics of our population include male and female, aged from birth to two years, and nationality as Saudi or non-Saudi, and the presence of registered height and weight. Exclusion criteria were all children who did not fit the inclusion criteria.

Sample size

The sample size was calculated by Raosoft (Seattle, WA, USA) sample size calculator with a 97% confidence level and 5% margins of error, and the minimum sample size required for this study was 283. We collected data from 142 participants. Purposive sampling was performed. The questionnaire was completed by research team members following interviews with the parents of the patients.

Study tool

An anonymous questionnaire was created using Google Forms. Initially, the authors designed the questionnaire, and it was validated by three experts in the field. Subsequently, the questionnaire was refined and adjusted based on their feedback and suggestions. The survey consisted of multiple-choice questions and dichotomous (Yes/No) questions. The final questionnaire comprised demographic data, anthropometric measurements, participants' medical conditions, mode of delivery, medical and natural history, and laboratory data. We used the MSD manual calculator to determine the percentages then the data was entered into the WHO growth chart.

Statistical analysis

Upon completion of data collection, the data were first entered into an Excel database (Microsoft, Redmond, WA, USA) for the purpose of cleaning, which included identifying and eliminating outliers, duplicates, and incomplete entries. After the data were cleaned, it was coded and transferred to SPSS software version 21 for analysis (IBM Corp., Armonk, NY, USA). Variables were presented as mean ± standard deviations (minimum - maximum) for parametric data and frequency (%) for categorized data. Comparison between groups was made using the Pearson Chi-Square test for categorized data and the unpaired Student “t” test for parametric data. Tests with a P-value < 0.05 are considered significant.

## Results

The current study’s objective is to determine the incidence of FTT and the various etiologies and risk factors responsible for it in a tertiary center in Jeddah, Saudi Arabia, between November 2023 and October 2024. A total of 142 patients aged 0 to two years were admitted to the pediatric ward during this period, of which 56.3% were male, while 43.7% were female. Patients were divided into four age groups, with those aged 0 to six months comprising 43.7%, followed by those between seven to 12 months (21.8%), 13 to 18 months (21.1%) and 19 to 24 months (12%). 70.4% of our patients were Saudi, while 29.6% were non-Saudi. The mean weight of patients was 6.61±2.87 kg, ranging from 1.8-17 kg, while the mean height was 64.49±13.06 cm, ranging from 44-129 cm. Of the patients, 68 (47.9%) reported a product of consanguineous marriage (Table 1).

**Table 1:** Demographic characteristics of all participants (n=142)

Characteristics		Value
Gender	Male	80 (56.3%)
	Female	62 (43.7%)
Age groups	0-6 months	62 (43.7%)
	7-12 months	31 (21.8%)
	13-18 months	30 (21.1%)
	19-24 months	17 (12.0%)
Nationality	Saudi	100 (70.4%)
	Non-Saudi	42 (29.6%)
Weight (kg)	Mean (±SD)	6.61± (2.87)
	Range	1.80-17.00
Height (cm)	Mean (±SD)	64.49± (13.06)
	Range	44-129
Consanguinity	Yes	68 (47.9%)
	No	74 (52.1%)

The age of delivery was mostly term (54.2%), then pre-term (15.5%) and post-term (2.1%), while 28.2% were unknown. Mode of delivery was mostly spontaneous vaginal delivery (51.4%) then cesarean section (CS) (43.0%), while 5.6% were not reported. Birth weight was normal in 50.0%, 21.8% reported low birth weight and 28.2% were unknown. Of all participants, 35.9% were previously admitted to a neonatal intensive care unit (NICU) or pediatric intensive care unit (PICU), 54.2% had congenital anomalies, and 58.5% had chronic illness. FTT was reported in 63 patients (44.4%), while 79 patients (55.6%) had no FTT. Hemoglobin levels were 11.03±2.74 gram/dl; mean corpuscular volume (MCV) levels were 79.39±9.17 (fL) and mean corpuscular hemoglobin (MCH) levels were 26.43±4.03 (pg) (Table 2).

**Table 2:** Antenatal and postnatal feature of children (n=142) NICU: Neonatal Intensive care unit, PICU: Pediatric intensive care unit, MCV: mean corpuscular volume, MCH: mean corpuscular hemoglobin

Features		Values
Age at delivery	Pre-term	22 (15.5%)
	Term	77 (54.2%)
	Post-term	3 (2.1%)
	Unknown	40 (28.2%)
Mode of delivery	Cesarean Section	61 (43.0%)
	Spontaneous vaginal delivery	73 (51.4%)
	Unknown	8 (5.6%)
Birth weight	Normal Birth Weight	71 (50.0%)
	Low Birth Weight	31 (21.8%)
	Unknown	40 (28.2%)
Previous NICU or PICU Admission	No	71 (50.0%)
	Yes	51 (35.9%)
	Unknown	20 (14.1%)
Congenital anomalies	No	65 (45.8%)
	Yes	77 (54.2%)
Chronic illness	No	59 (41.5%)
	Yes	83 (58.5%)
Failure to thrive	No	79 (55.6%)
	Yes	63 (44.4%)
Laboratory investigations	Hemoglobin (g/dl)	11.03±2.74 (6.3-20.9)
	MCV (fL)	79.39±9.17 (54.3-101.9)
	MCH (pg)	26.43±4.03 (16.5-35.4)

**Figure 1:**
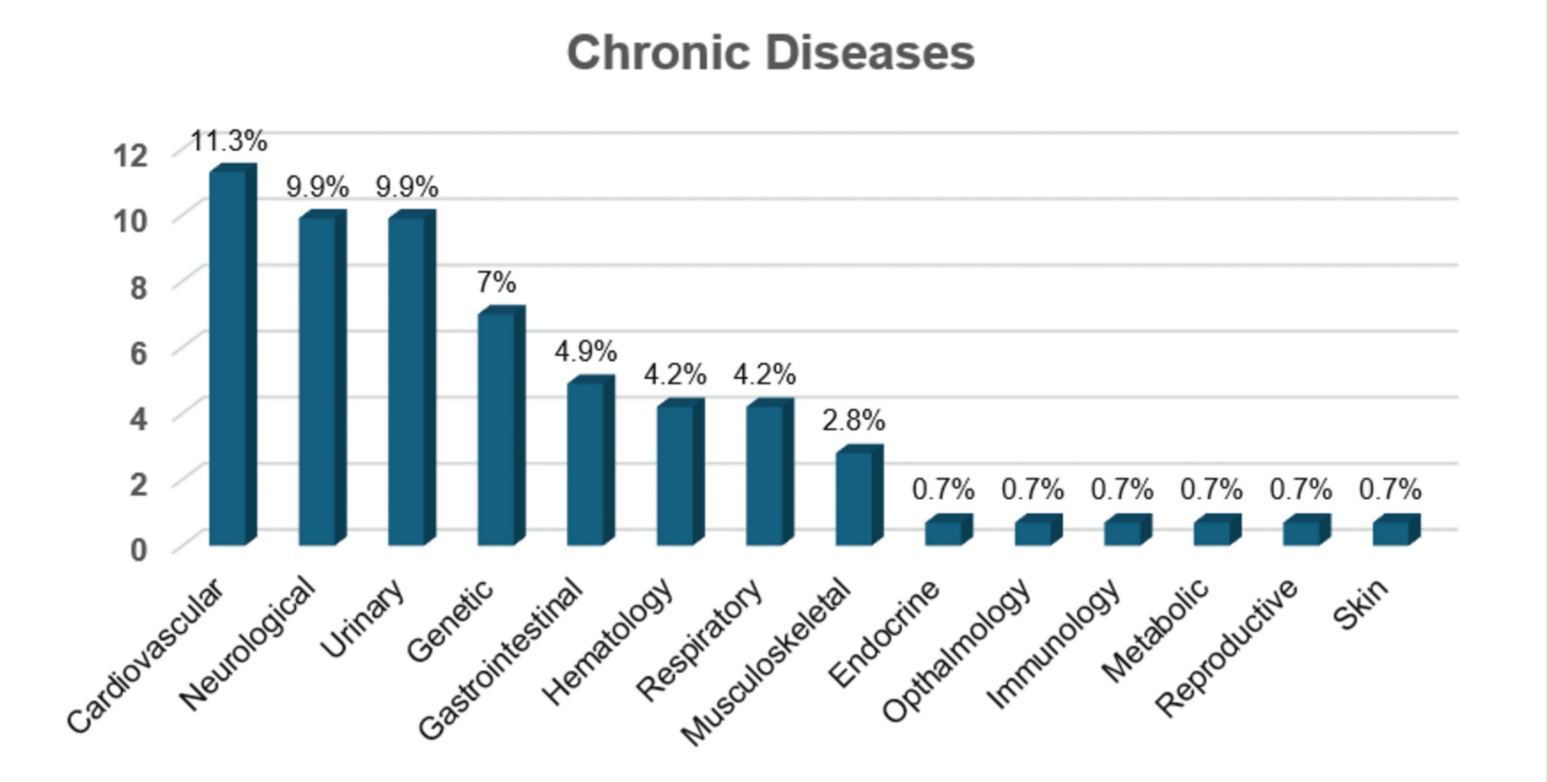
Percentage of chronic illness among participants

Regarding chronic illness, cardiovascular diseases have the highest prevalence (11.3%), followed closely by neurological and urinary diseases (both at 9.9%). Genetic conditions (7%) and gastrointestinal diseases (4.9%) also appear relatively common. Lower prevalence categories include endocrine (2.8%), ophthalmology, immunology, metabolic, reproductive, and skin diseases (each at 0.7%) (Figure 1).

Comparison of participants with and without failure to thrive according to demographic and clinical characteristics is shown in Table 3. Weight and height were significantly higher in no FTT patients than those with FTT (p<0.001 and p=0.012, respectively). Independent risk factor for failure to thrive were preterm births (p< 0.001), low birth weight (p<0.001), previous admission to NICU or PICU (p =0.004) and low hemoglobin contents (p=0.004).

**Table 3:** Demographic features and clinical characteristics in patients with and without failure to thrive. NICU: Neonatal Intensive care unit, PICU: Pediatric intensive care unit, MCV: mean corpuscular volume, MCH: mean corpuscular hemoglobin

Characteristics		Failure to thrive (n=63)	No failure to thrive (n=79)	Significance
Gender	Male	36 (57.1%)	44 (55.7%)	P= 0.863
	Female	27 (42.9%)	35 (44.3%)	
Age groups	0-6 months	34 (54.0%)	30 (38.0%)	P=0.076
	7-12 months	9 (14.3%)	22 (27.8%)	
	13-18 months	15 (23.8%)	15 (19.0%)	
	19-24 months	5 (7.9%)	12 (15.2%)	
Nationality	Saudi	42 (66.7%)	58 (73.4%)	P= 0.381
	Non-Saudi	21 (33.3%)	21 (26.6%)	
Weight (kg)	Mean (±SD)	5.16± (1.97)	(7.77±2.95)	P <0.001
	Range	1.80-8.66	2.84-17.00	
Height (cm)	Mean (±SD)	61.28± (14.32)	(67.14±11.36)	P= 0.012
	Range	44.00-129.00	47.00-90.00	
Consanguinity	Yes	33 (52.4%)	35 (44.3%)	P= 0.338
	No	30 (47.6%)	44 (55.7%)	
Age at delivery	Pre-term	2 (31.7%)	2 (2.5%)	P <0.001
	Term	30 (47.6%)	47 (59.5%)	
	Post-term	1 (1.6%)	2 (2.5%)	
	Unknown	12 (19.0%)	28 (35.4%)	
Mode of delivery	Cesarean Section	29 (46.0%)	32 (40.5%)	P= 0.775
	Spontaneous vaginal delivery	31 (49.2%)	42 (53.2%)	
	Unknown	3 (4.8%)	5 (6.3%)	
Birth weight	Normal Birth Weight	25 (39.7%)	46 (58.2%)	P <0.001
	Low Birth Weight	26 (41.3%)	5 (6.3%)	
	Unknown	12 (19.0%)	28 (35.4%)	
Previous NICU or PICU Admission	No	25 (39.7%)	46 (58.2%)	P =0.004
	Yes	32 (50.8%)	19 (24.1%)	
	Unknown	6 (9.5%)	14 (17.7%)	
Congenital anomalies	No	26 (41.3%)	39 (49.4%)	P=0.336
	Yes	37 (58.7%)	40 (50.6%)	
Chronic illness	No	24 (38.1%)	35 (44.3%)	P= 0.456
	Yes	39 (61.9%)	44 (55.7%)	
Laboratory investigations	Hemoglobin (g/dl)	10.17±2.10 (6.5-16.2)	11.91±3.05 (6.3-20.9)	P= 0.004
	MCV (fL)	79.06±10.26 (54.3-98.6)	79.73±8.02 (67.1-101.9)	P= 0.742
	MCH (pg)	26.14±4.09 (17.6-34.8)	26.72±4.01 (16.5-35.4)	P= 0.515

## Discussion

The findings of the study indicated that boys had a higher frequency of FTT compared to girls. This finding was supported by the study of Vaghari et al. [11]. On the other hand, a previous study done by Habibzadeh et al. showed that the incidence of growth failure in female infants was somewhat more than that in males [12]. A possible contributing factor would be biological differences. That is, boys may have higher energy requirements or different metabolic rates, which can affect growth.

Moreover, our study revealed a high incidence of FTT, at 44.4%, suggesting a considerable impact on the studied population. However, a study done by Alharbi A et al. (December 25, 2023) in Riyadh, Saudi Arabia, revealed that the prevalence of FTT is 26.7% [7]. The underlying cause can be attributed to negligence in clinical assessment. Although failure to thrive was identified in 44.4% of cases, it was documented and diagnosed by physicians in only 6.3% of instances, indicating a critical gap in clinical recognition and intervention for this serious condition.

Furthermore, our study demonstrated a notable association between NICU/PICU admissions and certain characteristics in children diagnosed with FTT. A significant 62.7% of those with prior NICU/PICU admissions were found to have FTT, suggesting that children requiring early intensive care face a higher risk of experiencing FTT later on.

This is supported by previous studies, which indicate that factors such as preterm birth, congenital anomalies, and neonatal complications are key drivers behind NICU admissions, especially in low-birth-weight infants, who are more likely to encounter developmental challenges, including FTT. For instance, a CDC report highlighted that approximately 15% of all NICU admissions are associated with low birth weight, a condition closely linked to later developmental issues, including FTT [13]. Additional research further illustrates that neonates with congenital anomalies often have complex care needs, with 38% of NICU admissions in a 2023 study being due to such anomalies, and these infants are more likely to experience growth failure and FTT as a result of their underlying conditions [14]. In a 2023 study, nearly 40% of preterm infants admitted to the NICU were found to suffer from long-term growth complications, including FTT [15]. These children often require prolonged intensive care, reflecting similar trends observed in our study population.

In addition to that, according to research, a low birth weight of 2±0.68 kg, which is much less than the typical range of 2.5-4.0 kg for term neonates, is linked to pediatric FTT [16]. Many studies have shown that low birth weight promotes developmental deficits [17].

Postnatal growth delays in low birth weight children can be caused by inadequate nutrition, sickness, and metabolic abnormalities [4]. A preterm delivery and intrauterine growth restriction (IUGR) might be the cause of the birth weight disparity in this group [4]. Very low birth weight (VLBW) newborns may experience developmental problems, and these circumstances may result in long-term growth failure and neurodevelopmental problems [17]. The study showed the critical role that early dietary treatment plays in repairing nutritional deficiencies and averting growth and development issues in low birth weight neonates [16]. These children are more prone to experience developmental problems if they don't receive the right medical attention and adequate nutrition [4]. The study highlights the necessity of taking early action and keeping a close eye on FTT infants to avoid developmental deficits [16]. The average birth weight of this sample, 2±0.68 kg, is consistent with evidence that links low birth weight to FTT and warns against ignoring the risk of long-term developmental issues [17].

Moreover, preterm infants are defined as less than 37 weeks of gestation [18]. Failure to thrive has always been related to preterm birth. A previous study found that preterm infants had a prevalence of 8.3% of FTT compared to term infants, which was 4.5% and with an even higher number of cases if the infant was born 32 weeks or less (19.0%) [19]. However, our study found that 90% of preterm infants had FTT, which is most likely due to not doing age correction for the preterm infants. Preterm babies are those that are born before 37 weeks of pregnancy, and as a result of their early birth, they frequently need a different developmental schedule than babies born at term. One technique to adjust for this discrepancy is through corrected age [20]. A child who does not meet developmental milestones may exhibit several physical and psychosocial traits that impede their ability to gain sufficient weight. Low birth weight babies (LBW: <2,500 g) have a higher chance of experiencing postnatal failure to thrive; in a similar study, 22% of preterm patients were diagnosed with FTT [21]. This proves that FTT has a strong association with preterm babies, which is consistent with our study.

Furthermore, according to our research, 42.1% of patients with FTT had no chronic illnesses, followed by cardiovascular disease (15.8%), neurological disorders (8.8%), and nephrological disorders (10.5%). The results suggest that there is no significant relationship between chronic diseases and FTT in this cross-sectional study. Cardiovascular disorders were the most common chronic condition identified, which is noteworthy since other studies, such as those by Ross et al. [1] and Alharbi et al. [7], have emphasized a broader spectrum of organic causes, including gastrointestinal and metabolic disorders. This variation may reflect regional or institutional differences in patient demographics or diagnostic criteria. These results highlight the need to consider local disease prevalence patterns when evaluating FTT cases. Nevertheless, the high percentage of patients without chronic diseases suggests that nonorganic factors, including environmental and psychosocial elements, may play a significant role in the etiology of FTT in this tertiary care center.

Study limitation

This study cannot be generalized for the whole of Saudi Arabia as it was conducted only in one center only so my recommendation in that there should be more studies regarding the incidence of FTT to estimate the true burden of this problem, other important limitation for this study is that the inclusion criteria was only inpatient which may overrepesent sicker or more sever cases of FTT, one of the important limitations are the small sample size may introduce bias; therefore, it should not be generalized to other hospitals. However, this represents the number of patients we were able to include within the study’s time frame and at the center where the study was conducted the information 

## Conclusions

Our study concluded a significant incidence of FTT among pediatric inpatients up to two years of age. Nearly half of our population had FTT. This might be due to the increasing number of patients covered by each resident. Notable risk factors include preterm births, ICU admissions, and an increasing number of congenital anomalies and diseases in the population. FTT might be considered a secondary problem, thus delaying the diagnosis and cure of the patient. These findings highlight the burden of FTT in KAUH, as well as the need for targeted interventions to address and reduce the incidence of FTT, as late diagnosis of FTT could delay treatment and increase the risk and complications of the condition.
